# Artificial intelligence for healthcare: restrained development despite impressive applications

**DOI:** 10.1186/s40249-025-01339-z

**Published:** 2025-07-20

**Authors:** Robert Bergquist, Laura Rinaldi, Xiao-Nong Zhou

**Affiliations:** 1 Geospatial Health, Ingerod, Brastad, Sweden; 2 Department of Veterinary Medicine and Animal Production, University of Naples Federico II, WHO Collaborating Centre for Diagnosis of Intestinal Helminths and Protozoa (WHOCC-ITA116), Via Delpino, 1, 80137 Naples, Italy; 3 National Institute of Parasitic Diseases at Chinese Center for Disease Control and Prevention (Chinese Center for Tropical Diseases Research); National Key Laboratory of Intelligent Tracking and Forecasting for Infectious Diseases; Key Laboratory on Parasite and Vector Biology, Ministry of Health; WHO Centre for Tropical Diseases; National Center for International Research on Tropical Diseases, Ministry of Science and Technology, Shanghai, 200025 China; 4 School of Global Health, Chinese Center for Tropical Diseases Research, Shanghai Jiao Tong University School of Medicine, Shanghai, 200025 China; 5 Hainan Center for Tropical Diseases Research (Sub-Center of Chinese Center for Tropical Diseases Research), Haikou, China

**Keywords:** Artificial intelligence, Healthcare, Climate change, Disease control, Diagnostics, Medical imaging

## Abstract

**Background:**

Artificial intelligence (AI) remains poorly understood and its rapid growth raises concerns reminiscent of dystopian narratives. AI has shown the capability of producing new medical content and improving management through optimization and standardization, which shortens queues, while its complete reliance on technical solutions threatens the traditional doctor-patient bond.

**Approach:**

Based on the World Economic Forum’s emphasis on the need for faster AI adoption in the medical field, we highlight current gaps in the understanding of its application and offer a set of priorities for future research. The historic review of AI and the latest publications point at barriers like complexity and fragmented regulations, while assisted analysis of big data offers new insights. AI’s potential in healthcare is linked to the breakthrough from rule-based computing, enabling autonomy through learning from experience and the capacity of reasoning. Without AI, protein folding would have remained unsolved, as emphasized by the Nobel-honored AlphaFold2 approach. It is expected that AI’s role in diagnostics, disease control, geospatial health and epidemiology will lead to similar progress.

**Conclusions:**

AI boosts efficiency, drives innovation, and solves complex problems but can also deepen biases and create security threats. Controlled progress requires industry collaboration leading to prompt acceleration of proper incorporation of AI into the health sphere. Cooperation between governments as well as both public and private sectors with a multi-actor approach is needed to effectively address these challenges. To fully harness AI’s potential in accelerating healthcare reform and shorten queues, while maintaining the compassionate essence of healthcare, a well-coordinated approach involving all stakeholders is necessary.

**Graphical abstract:**

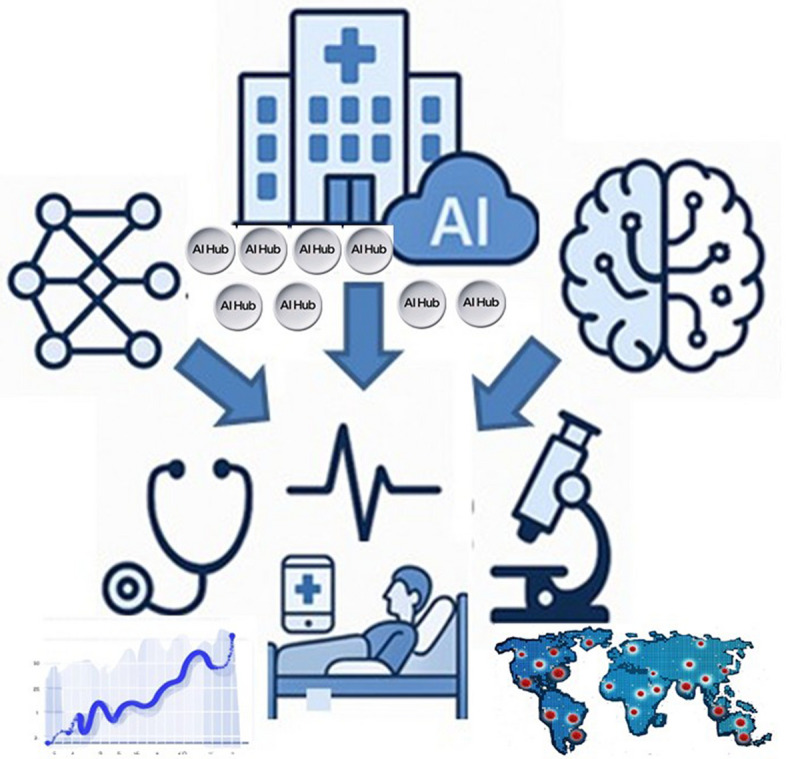

**Supplementary Information:**

The online version contains supplementary material available at 10.1186/s40249-025-01339-z.

## Background

Artificial intelligence (AI) has avoided the headlines until now, yet it has been with us for 75 years [1, 2]. Still, few understand what it really is and many feel uncomfortable about its rapid growth, with thoughts going back to the computer rebelling against the human crew onboard the spaceship heading out into the infinity of space in Arthur C. Clarke’s visionary novel “2001: a Space Odyssey” [3]. Just as in the novel, there is no way back since the human mind cannot continuously operate at an unwavering level of accuracy or simultaneous interact with different sections of large-scale information (Big Data), areas where AI excels. The World Economic Forum has made a call for a faster adoption of AI in the field of healthcare, a fact discussed at length in a very recent white-paper report [4] arguing that progress is not forthcoming as fast as expected despite the evident potential for growth and innovation at an all-time high and strong demand for new types of computer processors. Among the reasons mentioned for the slow uptake in areas dealing with healthcare are barriers, such as complexity deterring policymakers, and the risk for misaligned technical and strategic decisions due to fragmented regulations [4].

The growing importance of AI in the medical and veterinary fields strengthened by recent articles and editorials published in *The Lancet Digital Health* and *The Lancet* [5, 6] underlining actual and potential roles of AI in healthcare. We survey this wide spectrum highlighting current gaps in the understanding of AI and how its application can assist clinical work as well as support and accelerate basic research.

### AI technology development

#### From rules to autonomy

Before elaborating on these issues, some basic informatics about the technology that has moved AI to the fore is in order. In 1968, when both the film and the novel were released, only stationary, primitive computers existed. Rather than undergoing development in the preserve of large companies and academic institutions, they morphed into today’s public laptops, smartphones and wearable sensor networks. The next turn came with the gaming industry’s insatiable need for ultra-rapid action and life-like characters necessitating massively parallel computing, which led to switching from general-purpose, central processor units (CPUs) to specialized graphics processors (GPUs) and tensor processors (TPUs). Fuelled by this expansion of the processor architecture, neural networks, machine learning and elaborate algorithms capable of changing in conjunction with new data (meta-learning) were ushered in, with the rise of the power to understand and respond to human language through generative, pre-trained transformation (GPT) [7] showing the way forward. Breaking out of rule-based computing by the emergent capability of modifying internal settings, adapting to new information and understanding changing environments put these flexible systems, now referred to as AI, in the fast lane towards domains requiring high-level functionality. Computer systems adapted to a wide range of tasks, for which they were not explicitly programmed, could then be developed and launched into the public area as exemplified by automated industrial production, self-driving vehicles, virtual assistants and chatbots. Although lacking the imagination and versatility that characterize the human mind, AI can indeed perform tasks partly based on reasoning and planning that typically require human cognitive functions, and with enhanced efficiency and productivity.

#### Agent-based AI

Here, the agent is any entity that can perceive its environment, make decisions and act toward some goal, where rule-based AI has been replaced with proactive interaction. Agent-based AI generally uses many agents working separately to solve joint problems or even collaborating like a team. This approach was popularized by Wooldridge and Jennings in the 1990s, who described decentralized, autonomous AI systems capable of ‘meta-learning’ [8]. They felt that outside targets can be in sanitated and dealt with as computational objects, a methodology that has advanced the study of polarization, traffic flow, spread of disease, and similar phenomena. Although technology did not catch up with this vision until much later, AI today encompasses a vital area of active research producing powerful tools for simulating complex distributed and adaptive systems. The great potential of this approach for disease distributions and transmission dynamics may provide the insights needed to successfully control the neglected tropical diseases (NTDs) as well as dealing with other challenges in the geospatial health sphere [9]. The Internet of Things (IoT) [10], another example agent-based AI, represents the convergence of embedded sensors and software enabling collection and exchanging data with other devices and systems; however, operations are often local and do not necessarily involve the Internet.

**Fig. 1:**
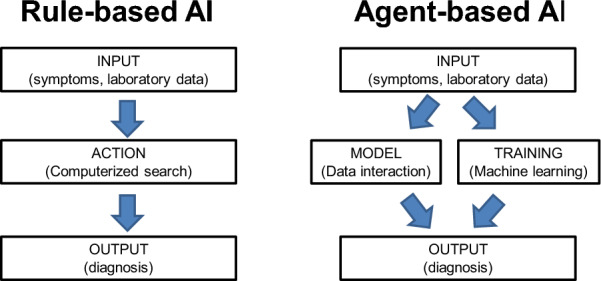
The research schemes of two AI’s approaches including Rule-based AI or Agent-based AI (AI refers artificial intelligence)

While the rule-based method follows a set of rules and therefore produces an outcome which is to some degree predictable, the two new components in the agent-based approach include the capability of learning from experience and testing various outcomes by one or several models. This introduces a level of reasoning, which allows for non-human choice, as schematically shown in Fig. 1.

### AI applications

#### Clinical applications

Contrary to common belief, a diagnostic program that today would be sorted under the heading AI was designed already 50 years ago at Stanford University, California, United States of America. The system, called MYCIN [11], was aimed to assist physicians with regard to bacterial blood infections. It was originally produced in book format, utilized a knowledge base of approximately 600 rules and operated through a series of questions to the user ultimately providing diagnosis and treatment recommendation. In the United States, similar approaches aimed at the diagnoses of bacterial infections appeared in the following decades but were not often used due to lack of computational power at the time. Today, on the other hand, this is no longer the limiting factor and AI is revolutionizing image-based diagnostics. In addition to the extensive use of AI-powered microscopy in parasitology, the spectrum includes both microscopic differentiation between healthy and cancerous tissue in microscope sections [12], as well as interpretations of graphs and videos from electrocardiography (EKG) [13], computer tomography (CT) [14, 15], magnet resonance imaging (MRI) [15] and ultrasonography [16]

Some AI-based companies are doing well, e.g., ACL Digital (https://www.acldigital.com/) that analyzes data from wearable sensors detecting heart arrhythmias, hypertension, sleep disorders; AIdoc (https://www.aidoc.com/eu/) whose platform evaluates clinical examinations and coordinates workflows beyond diagnosis; and the da Vinci Surgical System (https://en.wikipedia.org/wiki/Da_Vinci_Surgical_System), which has been used for various interventions, including kidney and hysterectiomy [17, 18]. However, others have failed, e.g., ‘Watson for Oncology’, launched by IBM for cancer diagnosis and optimized chemotherapy (https://www.henricodolfing.com/2024/12/case-study-ibm-watson-for-oncology-failure.html) and Babylon Health (https://en.wikipedia.org/wiki/Babylon_Health), a tele-health service that connected people to doctors via video calls, offered wholesale health promotion with high precision and virtual health assistants (Chatbots) that even remind patients to take medication. These final examples of AI-assisted medicine show that strong regulation is needed before this kind of assistance can be released for public use.

#### Basic research

The focus in the 2024 Nobel ceremony granted AI a central role: while the Physics Prize was awarded for the development of associative neural networks, the Chemistry Prize honored the breakthrough findings regarding how strings of amino acids fold into particular shapes [19]. This thorny problem was cracked by AlphaFold2, a robot based on deep-learning developed at DeepMind, a company that now belongs to Google’s parent Alphabet Inc. The finding that all proteins share the same folding process widened the research scope making it possible to design novel proteins with specific functions (synthetic biology), accelerate drug discovery and shed light on how diseases arise through mutations. The team that created this robot as its current sight on finding out how proteins interact with the rest of the cellular machinery. AlphaFold3, an updated version of the architecture generates accurate, three-dimensional molecular structures by pair-wise interaction between molecular components, which can be used to model how specific proteins work in union with other cell components exposing the details of protein interaction. These new applications highlight the exponential rise of AI’s significance for research in general and for medicine in particular.

The solution to the protein-folding problem not only reflects the importance of the training component but also demonstrates that AI is not as restricted as the human mind is when it comes to large realms of information (Big Data), which is needed for a large number of activities in modern society, such as autonomous driving, large-scale financial transactions as dealt with in banks on a daily basis. Big Data is common also in healthcare and it involves not only when dealing with hospital management and patient records, but also with large-sale diagnostic approaches. An academic paper, co-authored with clinicians and Google Research, investigated the reliability of diagnostic AI system, finding that machine learning reduced the number of false positives in a large mammography dataset by 25% (and also reached conclusions considerably faster), compared with the standard, clinical workflow without missing any true positives [20], a reassuring result.

#### Epidemiological surveillance

AI tools have been widely applied in epidemiological surveillance of vector-borne diseases. Due to vectors’ sensitivity to temperature and precipitation, the arthropod vectors are bellwether indicators, not only for the diseases they often carry but also for climate change. By gaining deeper insights into the complex interactions between climate, ecosystems and parasitic diseases with intricate life cycles, AI technologies assist by handling Big Data and even using reasoning to deal with obscure variations and interactions of climate and biological variables. To keep abreast of this situation, the connections between human, animal and environmental health not only demand data-sharing at the local level but also nationally and globally. This move towards the One Health/Planetary Health approach is highly desirable, and AI will unquestionably be needed for sustaining diligence with respect to the Big Data repositories required for accurate predictions of disease transmission, while AI-driven platforms can further facilitate real-time information exchange between stakeholders, optimize energy consumption and improve resource management for infections in animals and humans, in particular with regard to parasitic infections [21]. Proactive synergies between public health and other disciplines, such as ecology, genomics, proteomics, bioinformatics, sanitary engineering and socio-economy make the future medical agenda not only exciting and challenging, but also highly relevant globally.

In epidemiology, there has been a strong advance across the fields of medical and veterinary sciences [22], while previously overlooked events and unusual patterns now stand a better chance of being picked up by AI analysis of indirect methods, e.g., phone tracing, social media posts, news articles and health records. Technically less complex, but no less innovative operations are required to update the roadmap for elimination of the NTDs issued by the World Health Organization (WHO) [23]. The Expanded Special Project for the Elimination of Neglected Tropical Diseases (ESPEN) is a collaborative effort between the WHO regional office for Africa, member states and NTD partners. Its portal [24] offers visualization and planning tools based on satellite-generated imagery, climate data and historical disease patterns that are likely to identify high-risk areas for targeted interventions and allocate resources effectively. In this way, WHO’s roadmap for NTD elimination is becoming more data-driven, precise and scalable, thereby accelerating progress.

#### The publication records

Established as far back as 1993, *Artificial Intelligence Research* was the first journal specifically focused on AI, soon followed by an avalanche of similar ones (https://www.scimagojr.com/journalrank.php?category=1702). China, India and United States are particularly active in AI-related research. According to the *Artificial Intelligence Index Report 2024* [25], the total number of general AI publications had risen from approximately 88,000 in 2010 to more than 240,000 in 2022, with publications on machine learning increasing nearly sevenfold since 2015. If also conference papers and repository publications (such as arXiv) are included along with papers in both English and Chinese, the number rises to 900,000, with the great majority originating in China [26].

A literature search based solely on PubMed, carried out by the end of 2024 by us using “AI and infectious disease(s)” as search term resulted in close to 100,000 entries, while the term “Advanced AI and infectious disease(s)” only resulted in about 6600. The idea was to find the distintion between simpler, more rule-based applications and proper AI. Naturally, the results of this kind can be grossly misleading as information on the exact type of computer processor used, be it CPU, GPU or TPU, is generally absent and can only be inferred. Nevertheless, the much lower figure for “Advanced AI and infectious disease(s)” is an indication of the preference for less complex AI applications so far, i.e. work including spatial statistics and comparisons between various sets of variables vis-à-vis diseases, aiming at estimating distributions, hotspots, vector breeding sites, etc.

With as many as 100,000 medical publications found in the PubMed search, they clearly dominate in relation to the total of more than 240,000 AI-assisted research papers found up to 2022 [25]. The growing importance of this field is further strengthened by recent articles and editorials [27, 6]. Part of this interest is probably due to the wide spectrum of the medical and veterinary fields and AI’s potential in tracing and signalling disease outbreaks plus its growing role in surveillance that has led to a surge of publications on machine learning, offering innovative solutions to some of the most pressing challenges facing health research today [28].

## Conclusions

Healthcare reform, with special reference to improved coverage and shortening of queues, is urgently needed. The technology assisting this requires prompt acceleration of the incorporation of AI into the health sphere, but it must be strongly regulated to avoid bias. These challenges affect us all, requiring cooperation among all stakeholders, including governments as well as public and private sectors (Table S1). However, the shift from local epidemiological observations to a global dynamic approach without close collaboration with the computer industry has strained the production and sharing of results from epidemiological research and surveillance. If initiated, such collaboration could lead to specific computer systems adapted to dealing with the global field dynamically by knitting together results from ongoing control efforts in different endemic countries. Computer-assisted support has significantly contributed to advances in control and elimination of the NTDs, but projects still remain local , often focused on single countries and/or on single diseases. Here, AI could play an important role in reaching across borders, corralling stakeholders and sharing both data and information on resources more effectively. Although this is indeed ongoing in some places, a broader approach, faster advancement and better availability would be welcome.

## Supplementary Information

Additional file 1.

## Data Availability

Not applicable.

## Abbreviations

AI: Artificial intelligence
NTD: Neglected tropical diseases
CPUs: Central processor units
GPUs: Graphics processors
TPUs: Tensor processors
GPT: Generative, pre-trained transformation
EKG: Electrocardiography
CT: Computer tomography
MRI: Magnet resonance imaging
ESPEN: Expanded Special Project for the Elimination of Neglected Tropical Diseases
WHO: World Health Organization
