# Supplementary material for: Artificial intelligence for healthcare: restrained development despite impressive applications
Source: Infect Dis Poverty. 2025 Jul 20;14:72. doi: 10.1186/s40249-025-01339-z (PMC12276687; doi:10.1186/s40249-025-01339-z)
Supplement: Supplementary file 1 — Additional file 1. [file 40249_2025_1339_MOESM1_ESM.docx]

| **Supplementary Table S1.** **Examples of AI applications in healthcare and public health** | | | |
| --- | --- | --- | --- |
| **Fields** | **Explanation** | **Examples in applications** | **Challenges** |
| **Medical Diagnosis** | AI assists in diagnosing diseases through image analysis, pattern recognition and data interpretation. | MYCIN (bacterial infections) | Fragmented regulations |
|  |  | Microscopic tissue analysis | Complexity deterring policymakers |
|  |  | EKG, CT, MRI and ultrasonography interpretation | Misaligned technical and strategic decisions |
| **Epidemiological Surveillance** | AI analyzes large datasets to track disease spread, predict outbreaks and optimize resource allocation. | Vector-borne disease tracking (e.g., mosquitoes) | Need for global data-sharing |
|  |  | One Health approach | Integration of climate, ecological, and socio-economic data |
|  |  | ESPEN for NTD elimination | Unclear NTD transmission patterns in local settings |
| **Basic Research** | AI accelerates scientific discoveries, such as protein folding, drug discovery, and synthetic biology. | AlphaFold2/3 (protein folding) | Requires massive computational power |
|  |  | Drug discovery | Complexity of biological systems |
|  |  | Synthetic biology | Ethical concerns and need for cross-disciplinary collaboration |
| **Telehealth** | AI enables remote healthcare, health promotion and patient reminders via chatbots and virtual assistants. | Babylon Health (telehealth) | Public trust issues |
|  |  | AI chatbots for medication reminders | Regulatory challenges |
| **Healthcare Management** | AI improves hospital management, patient record handling and large-scale diagnostic approaches. | Mammography dataset analysis | Integration with existing systems |
|  |  | Hospital resource allocation | Data privacy concerns |
| **Public Health Interventions** | AI identifies high-risk areas, optimizes resource allocation, and supports targeted interventions for disease control. | WHO’s blueprint designed to achieve the roadmap for NTD elimination | Need for cross-border collaboration |
|  |  | Real-time information exchange platforms | Scalability of interventions |
| **Disease Modelling** | AI analyzes the interplay between climate, ecosystems and disease transmission to predict and mitigate risks. | Modelling vector-borne diseases | Integration of diverse datasets |
|  |  | Predicting hotspots of infectious diseases | Uncertainty in climate projections |

**Notes**: *AI refers artificial intelligence; NTD refers neglected tropical diseases; EKG refers* *electrocardiography; CT refers computer tomography; MRI refers magnet resonance imaging; ESPEN refers Expanded Special Project for* *the Elimination of Neglected Tropical Diseases; WHO refers World Health Organization.*
